# OA06.03. Spinal manipulative therapy, supervised rehabilitative exercise and home exercise for seniors with neck pain

**DOI:** 10.1186/1472-6882-12-S1-O23

**Published:** 2012-06-12

**Authors:** M Maiers, G Bronfort, R Evans, J Hartvigsen, K Svendsen, Y Bracha, C Schulz, K Schulz, R Grimm

**Affiliations:** 1 Northwestern Health Sciences University, Bloomington, USA; 2 University of Southern Denmark, Odense, Denmark; 3 Hennepin County Medical Center, Minneapolis, USA; 4 Berman Center for Outcomes and Clinical Research, Minneapolis, USA

## Purpose

Neck pain (NP) is a common condition in old age, leading to impaired functional ability and decreased independence. Spinal manipulation and exercise are common and effective treatments for the general NP population; however, their effectiveness among seniors is unknown. The primary aim of this randomized clinical trail was to assess the relative short- and long-term clinical effectiveness of spinal manipulative therapy (SMT) and supervised rehabilitative exercise (SRE), both in combination with and compared to home exercise (HE) alone, in NP patients 65 years and older.

## Methods

241 individuals age 65 and older with NP at least 12 weeks in duration were randomized to 12 weeks of treatment (above). The primary outcome was pain; secondary outcomes included disability, improvement, medication use, general health and satisfaction. Patient-rated outcome measures were collected via self-report questionnaires at baseline, 4, 12, 26, and 52 weeks post-randomization. Differences between the 3 groups were calculated for the short-term (weeks 4 and 12 data) and long-term (weeks 4, 12, 26, and 52 data) with a linear mixed model analysis.

## Results

In the short-term, SMT+HE demonstrated significantly greater reduction in pain compared to the other two groups. SMT+HE also showed greater improvement compared to HE alone. Both combined treatment groups were more satisfied at all time points. In the long-term, significant between group differences in both pain and improvement persisted in favor of SMT+HE over HE, but not SRE+HE. Participants in the combination therapies continued to report greater satisfaction with care than HE alone. There was less medication use among SMT+HE group compared to SRE+HE in the long term.

## Conclusion

In a senior population with chronic NP, SMT combined with home exercise results in less pain and greater improvement than home exercise alone in both the short-and long-term, and greater pain reduction than rehabilitative exercise plus home exercise in the short-term.

